# Examining the Combined Estimated Effects of Hearing Impairment and Depression on Cognitive Decline and Dementia

**DOI:** 10.1093/geroni/igab046.1848

**Published:** 2021-12-17

**Authors:** Danielle Powell, Willa Brenowitz, Kristine Yaffe, Frank Lin, Alden Gross, Jennifer Deal

**Affiliations:** 1 Johns Hopkins University, Baltimore, Maryland, United States; 2 UCSF, UCSF, California, United States; 3 Johns Hopkins University, Johns Hopkins University, Maryland, United States; 4 Johns Hopkins Bloomberg School of Public Health, Baltimore, Maryland, United States

## Abstract

Late-life depression is a comorbidity which may co-occur in older adults with hearing loss- each as prevalent and independent modifiable risk factors for dementia. We used data from 1,820 participants (74 ± 2.8 years, 38% Black race) from the Health Aging and Body Composition Study to test if the hearing loss-dementia/cognitive decline (Modified Mini Mental State Exam[3MS] and Digit Symbol Substitution[DSST]) relationship differed in hearing impaired participants who also had depressive symptoms. Depressive symptoms were defined as CES-D 10 ≥10) at one or more visits from years 1-5. Algorithmic incident dementia defined using medication use, hospitalizations and cognitive test scores. Audiometric hearing loss was measured at year 5 and categorized as normal/mild vs ≥moderate loss. In linear mixed models adjusted for demographic and clinical covariates, presence of both hearing loss and depressive symptoms (vs. having neither) was associated with faster rates of decline in 3MS (-0.30, 95% CI:-0.78, -0.19) and DSST (-0.35,95% CI:-0.67, -0.03) over 10 years of follow-up. Both hearing loss and depressive symptoms (vs. neither) was associated with increased risk (hazard ratio (HR):2.91, 95%CI: 1.59, 5.33) of incident dementia in multivariable-adjusted Cox proportional hazards models. Comorbid conditions among hearing impaired older adults should be considered and may aid in dementia prevention and management strategies.

